# Supplementary material for: Co-Occurrence and Cooperation between Comammox and Anammox Bacteria in a Full-Scale Attached Growth Municipal Wastewater Treatment Process
Source: Environ Sci Technol. 2023 Mar 13;57(12):5013–23. doi: 10.1021/acs.est.2c09223 (PMC10061930; doi:10.1021/acs.est.2c09223)
Supplement: Supplementary file 1 — es2c09223_si_001.pdf [file es2c09223_si_001.pdf]

SUPPORTING INFORMATION

Co-occurrence and cooperation between comammox and anammox bacteria in a full-scale attached growth municipal wastewater treatment process.

Katherine Vilardi[1#], Irmarie Cotto[2#], Megan Bachmann[3], Mike Parsons[3], Stephanie Klaus[3], Christopher Wilson[3], Charles B. Bott[3], Kelsey J. Pieper[1], Ameet J. Pinto[2*]

[1] Department of Civil and Environmental Engineering, Northeastern University, 360 Huntington Avenue, Boston, Massachusetts, 02115, USA
[2] School of Civil and Environmental Engineering, Georgia Institute of Technology, 311 Ferst Drive Atlanta, Georgia 30318, USA
[3] Hampton Roads Sanitation District, 1434 Air Rail Avenue, Virginia Beach, Virginia 23455, USA.

*Corresponding author: Ameet Pinto (ameet.pinto@ce.gatech.edu)
[#] Both authors contributed equally to this manuscript.

DESCRIPTION OF SUPPORTING INFORMATION

Section SI-1. Preparation of Batch Assay Set-up and Experiments

Section SI-2. Sample characterization and total solids measurements

Section SI-3. Sample collection and processing for full-scale experiments

Section SI-4. In situ rate calculations

Table SI-1: Rates obtained from triplicate aerobic ammonia and nitrite oxidation, and anaerobic ammonia oxidation batch assays

Table SI-2: Total number of raw reads and reads remaining in sample fastq files after quality filtering with fastp

Table SI-3: Set of nitrifier MAGs recovered from samples taken from June 2017 to June 2018 and one sample (Sample2) from December 2021 after dereplication.

Table SI-4: Publicly available *Nitrospira*, *Brocadia* and *Nitrosomonas* genomes downloaded from NCBI.

Table SI-5: Percent of genomes covered by reads (breadth) in six metagenomes.

Table SI-6: Relative abundances in reads per kilobase million (RPKM) of MAGs with average genome coverage higher than 50%.

Figure SI-1: Specific rates for ammonia oxidation, nitrate production, and loss of total inorganic nitrogen for the suspended solids.

Figure SI-2: Chemical and physical process parameters measured along the transect of the IFAS system.

Figure SI-3: Nitrogen species as a percent of influent total inorganic nitrogen along the transect of the IFAS system.

Figure SI-4: Percent of ammonia and total inorganic nitrogen removal in the aerobic zone at each DO set point.

Figure SI-5: Specific rates for aerobic and anaerobic nitrogen biotransformation in the aerobic zone of the full-scale IFAS system during DO set point experiments

Section SI-1. Preparation of Batch Assay Set-up and Experiments

Stock solutions were prepared as 10,000 mg NH_4-N/L (using ammonium chloride) and 10,000 mg NO_2-N/L (using sodium nitrite). Prior to conducting experiments, the IFAS media was collected from the aerobic zone and washed overnight in secondary clarifier effluent to remove loosely associated biomass. 1 L glass beakers were set up on stir plates with a magnetic bar placed inside along with an aeration stone connected to an aquarium pump to provide aeration for aerobic assays. After washing with secondary effluent, 40 pieces of IFAS media were added to the beakers with 900 mL of secondary clarifier effluent for the attached phase assays while 900 mL mixed liquor were added to beakers for suspended phase assays. The aquarium pump was turned on to provide aeration for aerobic experiments with pump settings modified based on DO measurements to achieve a DO concentration between 6-8 mg/L. Alkalinity and pH in all beakers were measured before the experiments and adjusted using the sodium bicarbonate stock solution to ensure adequate alkalinity for nitrification. Blank controls were established for each condition using 900 mL of filtered water.

Section SI-2. Sample characterization and total solids measurements

DO concentrations and temperature were measured directly at each sampling location in the full-scale IFAS system using a probe (IPM insiteIG with PortaCaddie) submerged in the wastewater. pH was measured using the HACH Company Pocket Pro+ pH meter (Cat. No. 9532000) immediately after sample collection. Duplicate samples of mixed liquor were filtered at all locations in the field through 0.45 μM syringe filters for chemical analysis. Hach Company TNT Vials were used to determine concentrations of ammonia (TNT832, TNT831), nitrite (TNT839, TNT840), nitrate (TNT835), chemical oxygen demand (TNT821, TNT820), and total alkalinity (TNT870) of the filtered aqueous samples from both full-scale and intrinsic kinetic experiments. All samples were analyzed on a HACH DR3900 photospectrometer (Cat. No. LPV440.99.00002). IFAS media and suspended solids from both ends of the aerobic zone were collected on the sampling days to measure total solids (TS) and total suspended solids (TSS). Suspended solids and IFAS media were also collected from both ends of the aerobic zone for DNA extraction for microbial characterization.

Three IFAS pieces and suspended solids samples were taken from two aeration zones (beginning and end) on three different sampling dates. The suspended solids concentration (g/L) was measured using Standard Method 2540D. All IFAS pieces (n=18) were baked overnight at 105 °C and then weighed before scrubbing off all biomass with a 2 N H_2SO_4 solution and small bristle brush. The clean IFAS pieces were dried overnight at 105 °C and then weighed. The mass of total solids (TS) on individual pieces were obtained by subtracting the mass of the cleaned IFAS piece from the mass recorded for dried attached biomass. The average mass of TS per media piece for each sampling date was then multiplied by 40 (number of IFAS pieces used in each assay) to estimate the total mass of attached biomass in batch assays for the attached phase. The average concentration of total attached biomass in grams per liter was estimated by dividing the average total mass of attached biomass (g) by 0.9 L (volume of secondary clarifier effluent used in batch assays). Total attached biomass in the aerobic zone of the treatment train was estimated by multiplying the average mass of attached biomass obtained from all 18 pieces by the number of IFAS pieces in the aerobic zone (~114155600). The average concentration of total attached biomass (g/L) was estimated by dividing the aforementioned average (g) by the tank volume, 984207 L.

Section SI-3. Sample collection and processing for full-scale experiments

The six sampling locations selected included the anoxic zone (n=3, one per stage for R1, R2, and R3), aerobic zone (n=2, one in the beginning (R4_Z1) and one at the end (R4_Z4)), and deaeration (n=1, R5) (Figure 1 main manuscript). Samples were collected using a long handle scooper approximately six hours after the DO concentration was modified in the aerobic zone, and only one DO setting was applied per day. In the field, IFAS media was stored by cutting pieces with a sterile razor blade and placed two per tube into 15 mL falcon tubes for DNA extraction. Both IFAS media and suspended solids samples for DNA extraction were placed in a -20 °C freezer at the treatment plant until they were shipped on dry ice to Northeastern University, where they remained in a -80 °C freezer until extraction. Samples were taken at five designated sampling locations along the transect on days when the DO was set to 2, 4, and 6 mg/L for measuring volatile fatty acids (VFAs). Seven VFAs, including acetic, butyric, caproic, isobutyric, isovaleric, propionic and valeric acid, were surveyed using standard method SM5560D, but only acetic acid was detected above the limit of quantification (LOQ = 5 mg/L) in any of the wastewater samples.

Section SI-4. In situ rate calculations

In situ rates of ammonia oxidation, nitrite oxidation, nitrate production, and loss of total inorganic nitrogen in the aerobic zone were calculated using the following equations:

Ammonia oxidation rate (rNH_3) = (($NH_{3,in}$ - $NH_{3,eff}$)/Aerobic HRT)/TS (1)

Nitrate production rate (rNO_3) = (($NO_{3,eff}$ - $NO_{3,in}$)/Aerobic HRT)/TS (2)

Total inorganic nitrogen removal rate (rTIN) = ((($NH_{3,in}$ + $NO_{2,in}$ + $NO_{3,in}$) - ($NH_{3,eff}$ + $NO_{2,eff}$ + $NO_{3,eff}$))/ Aerobic HRT)/TS (3)

Anaerobic ammonia oxidation rate ($rNH_{3,ANA}$) = rTIN/2.04 (4)

Anaerobic nitrite reduction rate ($rNO_{2,ANA}$) = 1.32(rTIN/2.04) (5)

Anaerobic nitrate production rate ($rNO_{3,ANA}$) = 0.26(rTIN/2.04) (6)

Aerobic ammonia oxidation ($rNH_{3,AER}$) = rNH_3 - $rNH_{3,ANA}$ (7)

Aerobic nitrate production rate ($rNO_{3,AER}$) = rNO_3 - $rNO_{3,ANA}$ (8)

Average concentrations (mg-N/L) for ammonia, nitrite, and nitrate were obtained from duplicates samples collected from the influent to the aerobic zone (subscript "in") at the final anoxic zone at R3 and effluent from the aerobic zone (subscript "eff") at R4_Z4. Total inorganic nitrogen (mg/L) was determined by the summation of ammonia, nitrite, and nitrate concentrations measured at R3 and R4_Z4. The hydraulic retention time (HRT) of the aerobic zone expressed in hours was calculated by dividing the volume of the aerobic tank by the flowrate across the aerobic zone on day of sampling. Estimates for anaerobic ammonia oxidation ($rNH_{3,ANA}$), nitrite reduction ($rNO_{2,ANA}$) and nitrate production ($rNO_{3,ANA}$) rates were calculated using the constants derived from anammox reaction stoichiometry corresponding to 2.04 (1 mol $1NH_4^+$ consumed produces 1.02 mol N_2: $1NH4^+ + 1.32NO_2 \rightarrow 1.02N_2 + 0.26NO_3^-$) of nitrogen (as N), 0.26 mol of nitrate (as N) and 1.32 mol of nitrite (as N) per mole of ammonium consumed. All rates are expressed as specific rates (mg-N/g TS-hr) calculated by dividing the *in situ* rates by the estimated concentration of total attached biomass in the aerobic zone.

Table SI-1: Rates obtained from triplicate aerobic ammonia and nitrite oxidation, and anaerobic ammonia oxidation batch assays for the attached growth phase.

Assay	Rate type	Average rate (mg-N/g TS-h)	Standard deviation
Aerobic ammonia oxidation	Ammonia removal	1.75	0.08
	Nitrate production	1.15	0.09
	NOx production	1.22	0.09
	TIN loss	0.53	0.10
Aerobic nitrite oxidation	Nitrite oxidation	3.23	0.36
	Nitrate production	3.16	0.40
	TIN loss	0.10	0.03
Anaerobic ammonia oxidation	Ammonia removal	1.13	0.17
	Nitrite reduction	1.36	0.45
	Nitrate production	0.36	0.18
	TIN loss	2.15	0.55

TIN: total inorganic nitrogen

Table SI-2: Total number of raw reads and reads remaining in sample fastq files after quality filtering with fastp

Sample	Raw	Filtered
1	251564616	243864532
2	209058090	202372028
3	208199202	201819776
4	220372610	212874004
5	192953414	186805330
6	198420028	191168754

Table SI-3: Set of nitrifier MAGs recovered from samples taken from June 2017 to June 2018 and one sample (Sample2) from December 2021 after dereplication.

Genome	Completeness	Redundancy	Length	Contigs	GC content	GTDB Taxonomy
Brocadia _bin.281*	92.00	0.00	2868031	125	42.2	d__Bacteria;p__Planctomycetota;c__Brocadiae;o__Brocadiales;f__Brocadiaceae;g__Brocadia;s__Brocadia sapporoensis
Brocadia_bin.357*	90.00	0.00	2601165	241	43.5	d__Bacteria;p__Planctomycetota;c__Brocadiae;o__Brocadiales;f__Brocadiaceae;g__Brocadia;s__Brocadia pituitae
JAMMSM_AOB_1	94.12	9.80	2926721	166	47.1	d__Bacteria;p__Proteobacteria;c__Gammaproteobacteria;o__Burkholderiales;f__Nitrosomonadaceae;g__Nitrosomonas;s__
JAMMSM_AOB_2	100.00	2.63	2859940	22	48.8	d__Bacteria;p__Proteobacteria;c__Gammaproteobacteria;o__Burkholderiales;f__Nitrosomonadaceae;g__Nitrosomonas;s__
JAMMSM_AOB_3	100.00	0.00	2882584	21	43.0	d__Bacteria;p__Proteobacteria;c__Gammaproteobacteria;o__Burkholderiales;f__Nitrosomonadaceae;g__Nitrosomonas;s__
JAMMSM_AOB_4	96.08	0.00	1957621	82	41.8	d__Bacteria;p__Proteobacteria;c__Gammaproteobacteria;o__Burkholderiales;f__Nitrosomonadaceae;g__Nitrosomonas;s__
JAMMSM_AOB_5	98.04	5.26	2568442	19	42.5	d__Bacteria;p__Proteobacteria;c__Gammaproteobacteria;o__Burkholderiales;f__Nitrosomonadaceae;g__Nitrosomonas;s__
JAMMSM_AOB_6	92.16	1.96	2031342	65	47.6	d__Bacteria;p__Proteobacteria;c__Gammaproteobacteria;o__Burkholderiales;f__Nitrosomonadaceae;g__Nitrosomonas;s__
JAMMSM_AOB_7	94.12	2.63	3066737	73	43.6	d__Bacteria;p__Proteobacteria;c__Gammaproteobacteria;o__Burkholderiales;f__Nitrosomonadaceae;g__Nitrosomonas;s__
JAMMSM_AOB_8	98.04	2.63	2970458	91	45.0	d__Bacteria;p__Proteobacteria;c__Gammaproteobacteria;o__Burkholderiales;f__Nitrosomonadaceae;g__Nitrosomonas;s__

JAMMSM_AOB_9	78.43	1.96	2167635	21	46.2	d__Bacteria;p__Proteobacteria;c__Gammaproteobacteria;o__Burkholderiales;f__Nitrosomonadaceae;g__Nitrosomonas;s__
JAMMSM_CMX_1	94.12	1.96	4065392	23	54.8	d__Bacteria;p__Nitrospirota;c__Nitrospiria;o__Nitrospirales;f__Nitrospiraceae;g__Nitrospira;s__Nitrospira sp002254365GCA_002254365.1
JAMMSM_CMX_2	86.27	1.96	2629561	22	55.3	d__Bacteria;p__Nitrospirota;c__Nitrospiria;o__Nitrospirales;f__Nitrospiraceae;g__Nitrospira;s__
JAMMSM_NOB_2	90.20	0.00	2828782	50	58.9	d__Bacteria;p__Nitrospirota;c__Nitrospiria;o__Nitrospirales;f__Nitrospiraceae;g__Nitrospira_D;s__
JAMMSM_NOB_4	100.00	0.00	3829071	5	58.7	d__Bacteria;p__Nitrospirota;c__Nitrospiria;o__Nitrospirales;f__Nitrospiraceae;g__Nitrospira_A;s__
JAMMSM_NOB_5	70.59	2.63	2923168	59	58.9	d__Bacteria;p__Nitrospirota;c__Nitrospiria;o__Nitrospirales;f__Nitrospiraceae;g__Nitrospira_A;s__
JAMMSM_NOB_6	88.24	7.89	2856077	119	58.5	d__Bacteria;p__Nitrospirota;c__Nitrospiria;o__Nitrospirales;f__Nitrospiraceae;g__Nitrospira_A;s__
JAMMSM_NOB_7	84.31	0.00	2879078	61	57.6	d__Bacteria;p__Nitrospirota;c__Nitrospiria;o__Nitrospirales;f__Nitrospiraceae;g__Nitrospira_D;s__
Nitrospira_bin.184*	94.00	6.00	4812459	231	59.4	d__Bacteria;p__Nitrospirota;c__Nitrospiria;o__Nitrospirales;f__Nitrospiraceae;g__Nitrospira_A;s__
Nitrospira_bin.465*	90.00	2.00	3222716	235	58.4	d__Bacteria;p__Nitrospirota;c__Nitrospiria;o__Nitrospirales;f__Nitrospiraceae;g__Nitrospira_A;s__Nitrospira_A defluvii_A

*MAGs recovered from this study

Table SI-4: Publicly available *Nitrospira*, *Brocadia* and *Nitrosomonas* genomes downloaded from NCBI. Genomes previously assembled from JRTP by Cotto et al 2023 are shown in bold.

**Reference genomes**	**Accession number**
Leptospirillum rubarum	GCA_000205145.2
Candidatus Nitrospira defluvii	GCA_000196815.1
Candidatus Nitrospira inopinata	GCF_001458695.1
Candidatus Nitrospira kreftii isolate	GCA_014058405.1
Candidatus Nitrospira nitrificans	GCF_001458775.1
Candidatus Nitrospira nitrosa	GCF_001458735.1
Nitrospira bin RBC001	GCA_902500755.1
Nitrospira bin RBC003	GCA_902500695.1
Nitrospira bin RBC035	GCA_902501035.1
Nitrospira bin RBC042	GCA_902500795.1
Nitrospira bin RBC044	GCA_902500705.1
Nitrospira bin RBC048	GCA_902500785.1
Nitrospira bin RBC069	GCA_902500775.1
Nitrospira bin RBC073	GCA_902500995.1
Nitrospira bin RBC083	GCA_902500745.1
Nitrospira bin RBC085	GCA_902500715.1
Nitrospira bin RBC093	GCA_902500825.1
Nitrospira bin RBC100	GCA_902500805.1
Nitrospira japonica	GCF_900169565.1
Nitrospira lenta	GCF_900403705.1
Nitrospira moscoviensis	GCF_001273775.1
Nitrospira sp. AMP bin1	GCA_018242825.1
Nitrospira sp. AMP bin2	GCA_018242805.1
Nitrospira sp. bin75	GCA_002238765.1
Nitrospira sp. CG24A	GCA_002869925.2
Nitrospira sp. CG24B	GCA_002869845.2
Nitrospira sp. CG24C	GCA_002869885.2
Nitrospira sp. CG24D	GCA_002869855.2
Nitrospira sp. CG24E	GCA_002869895.2
Nitrospira sp. Ga0074138	GCA_001464735.1
Nitrospira sp. KAN bin1	GCA_018242765.1
Nitrospira sp. KAN bin2	GCA_018242725.1
Nitrospira sp. KAN bin3	GCA_018242735.1
Nitrospira sp. LK265	GCA_011090395.1
Nitrospira sp. LK70	GCA_011090425.1
Nitrospira sp. MAG 137	GCA_009594955.1

Nitrospira sp. MAG 149	GCA_009594935.1
Nitrospira sp. MAG 237	GCA_009595005.1
Nitrospira sp. MAG 242	GCA_009594945.1
Nitrospira sp. MAG 245	GCA_009594825.1
Nitrospira sp. MAG 246	GCA_009594995.1
Nitrospira sp. MAG 248	GCA_009594835.1
Nitrospira sp. MAG 266	GCA_009594865.1
Nitrospira sp. MAG 275	GCA_009594855.1
Nitrospira sp. MAG 79	GCA_902500715.1
Nitrospira sp. ND1	GCF_900170025.1
Nitrospira sp. OLB3	GCA_001567445.1
Nitrospira sp. RCA	GCA_005239465.1
Nitrospira sp. RCB	GCA_005239475.1
Nitrospira sp. RSF1 1	GCA_005116965.1
Nitrospira sp. RSF11 1	GCA_005116945.1
Nitrospira sp. RSF12 1	GCA_005116955.1
Nitrospira sp. RSF127 1	GCA_005116935.1
Nitrospira sp. RSF13 1	GCA_005116865.1
Nitrospira sp. RSF151 1	GCA_005116795.1
Nitrospira sp. RSF3 1	GCA_005116835.1
Nitrospira sp. RSF48 1	GCA_005116775.1
Nitrospira sp. RSF5 1	GCA_005116895.1
Nitrospira sp. RSF6 1	GCA_005116885.1
Nitrospira sp. RSF7 1	GCA_005116825.1
Nitrospira sp. RSF8 1	GCA_005116815.1
Nitrospira sp. RSF9 1	GCA_005116745.1
Nitrospira sp. SCGC	GCA_001644405.1
Nitrospira sp. SCN 59-13	GCA_001724505.1
Nitrospira sp. SG-bin1	GCA_002083365.1
Nitrospira sp. SG-bin2	GCA_002083405.1
Nitrospira sp. ST-bin4	GCA_002083565.1
Nitrospira sp. ST-bin5	GCA_002083555.1
Nitrospira sp. TMP bin1	GCA_018242685.1
Nitrospira sp. TMP bin2	GCA_018242705.1
Nitrospira sp. TMP bin3	GCA_018242665.1
Nitrospira sp. UW-LDO-01	GCA_002254365.1
Nitrospira sp. UW-LDO-02	GCA_002254325.1
Nitrospira sp. WS110	GCA_011090365.1
GRE_NOB_1	GCA_024281635.1

GRE_NOB_2	GCA_024281645.1
NEU_NOB_1	GCA_024281525.1
NEU_NOB_2	GCA_024281465.1
**JAMMSM_NOB_1**	**GCA_024281615.1**
**JAMMSM_NOB_2**	**GCA_024281595.1**
**JAMMSM_NOB_3**	**GCA_024281555.1**
**JAMMSM_NOB_4**	**GCA_024281575.1**
**JAMMSM_NOB_5**	**GCA_024281515.1**
**JAMMSM_NOB_6**	**GCA_024281495.1**
**JAMMSM_NOB_7**	**GCA_024281455.1**
Nitrosococcus oceani ATCC 19707	GCA_000012805.1
Nitrosomonas aestuarii strain Nm36 Ga0181080 101	GCF_003046585.1
Nitrosomonas aestuarii strain Nm69	GCF_900114305.1
Nitrosomonas communis strain Nm110	GCF_900106545.1
Nitrosomonas communis strain Nm2 chromosome	GCF_001007935.1
Nitrosomonas communis strain Nm44	GCF_900114745.1
Nitrosomonas cryotolerans strain Nm55	GCF_900115725.1
Nitrosomonas europaea isolate OLB2	GCA_001567435.1
Nitrosomonas europaea strain ATCC 25978	GCF_900167395.1
Nitrosomonas eutropha C91	GCF_000014765.1
Nitrosomonas eutropha strain Nm19 Ga0181055 101	GCF_003046355.1
Nitrosomonas eutropha strain Nm24	GCF_900116685.1
Nitrosomonas eutropha strain Nm38	GCF_900106875.1
Nitrosomonas halophila strain Nm1	GCF_900107165.1
Nitrosomonas marina strain Nm22	GCF_900110145.1
Nitrosomonas marina strain Nm71	GCF_900111605.1
Nitrosomonas mobilis isolate 1	GCF_900103035.1
Nitrosomonas nitrosa strain Nm90 Ga0181054 101	GCF_003051105.1
Nitrosomonas oligotropha strain Nm49 Ga0181079 101	GCF_003050805.1
Nitrosomonas oligotropha strain Nm76	GCF_900110385.1
Nitrosomonas sp. AL212	GCF_000175095.2
Nitrosomonas sp. APG5	GCF_003011925.2
Nitrosomonas sp. Is79A3	GCF_000219585.1
Nitrosomonas sp. isolate F1-120-MAGs074	GCA_005779435.1
Nitrosomonas sp. isolate UBA8640	GCA_003514765.1
Nitrosomonas sp. Nm120 Ga0180021 11	GCF_003664085.1
Nitrosomonas sp. Nm132	GCF_900100485.1

Nitrosomonas sp. Nm134 Ga0181067 101	GCF_004345375.1
Nitrosomonas sp. Nm141 Ga0181066 101	GCF_003046215.1
Nitrosomonas sp. Nm143 Ga0207393 101	GCF_003268955.1
Nitrosomonas sp. Nm166	GCF_900112825.1
Nitrosomonas sp. Nm33	GCF_900107265.1
Nitrosomonas sp. Nm34	GCF_900113925.1
Nitrosomonas sp. Nm51	GCF_900111165.1
Nitrosomonas sp. Nm58	GCF_900107335.1
Nitrosomonas sp. Nm86 Ga0181061 101	GCF_004340695.1
Nitrosomonas sp. UBA6494	GCA_002435335.1
Nitrosomonas stercoris KYUHI-S	GCA_006742785.1
Nitrosomonas ureae strain Nm10	GCF_900105875.1
Nitrosomonas ureae strain Nm13	GCF_900108305.1
Nitrosomonas ureae strain Nm15	GCF_900206265.1
Nitrosomonas ureae strain Nm42	GCF_900215345.1
Nitrosomonas ureae strain Nm4 Ga0181063 1001	GCF_003051045.1
Nitrosomonas ureae strain Nm5 Ga0181075 101	GCF_003201725.1
Nitrosomonas ureae strain Nm9	GCF_900111055.1
Proteobacteria bacterium SG bin4 1077	GCA_002083395.1
Proteobacteria bacterium ST bin16 1024	GCA_002083595.1
uncultured beta proteobacterium isolate AOB	GCA_900696615.1
GRE_AOB_1	GCA_024282195.1
GRE_AOB_2	GCA_024282035.1
GRE_AOB_3	GCA_024282095.1
GRE_AOB_4	GCA_024282045.1
NEU_AOB_1	GCA_024281765.1
NEU_AOB_2	GCA_024281695.1
NEU_AOB_3	GCA_024281715.1
NEU_AOB_4	GCA_024281675.1
**JAMMSM_AOB_1**	**GCA_024282015.1**
**JAMMSM_AOB_2**	**GCA_024281955.1**
**JAMMSM_AOB_3**	**GCA_024281995.1**
**JAMMSM_AOB_4**	**GCA_024281935.1**
**JAMMSM_AOB_5**	**GCA_024281825.1**
**JAMMSM_AOB_6**	**GCA_024281815.1**
**JAMMSM_AOB_7**	**GCA_024281795.1**
**JAMMSM_AOB_8**	**GCA_024281735.1**
**JAMMSM_AOB_9**	**GCA_024281755.1**
Pirellula staleyi	GCA_000025185.1

Candidatus Jettenia caeni	GCA_000296795.1
Candidatus Kuenenia stuttgartiensis	GCA_000315095.1
Candidatus Scalindua brodae isolate RU1	GCA_000786775.1
Candidatus Brocadia sinica JPN1	GCA_000949635.1
Candidatus Brocadia fulgida isolate RU1	GCA_000987375.1
Candidatus Scalindua rubra isolate BSI-1	GCA_001723765.1
Candidatus Brocadia caroliniensis isolate 26THWARD	GCA_002009475.1
Candidatus Brocadia sp. UTAMX2	GCA_002050315.1
Candidatus Brocadia sp. UTAMX1	GCA_002050325.1
Candiadtus Scalindua japonica husup-a2	GCA_002443295.1
Candidatus Scalindua rubra isolate Ru_enrich_A1	GCA_002632345.1
Candidatus Kuenenia stuttgartiensis isolate Ru_enrich_A3	GCA_002632395.1
Candidatus Brocadia sp. R4W10303	GCA_002848945.1
Candidatus Brocadia sp. isolate AMX2	GCA_003577195.1
Candidatus Brocadia sp. isolate AMX1	GCA_003577215.1
Candidatus Brocadia sp. BROELEC01	GCA_004282735.1
Candidatus Scalindua sp. SCAELEC01	GCA_004282745.1
Candidatus Scalindua sp. AMX11	GCA_004351875.1
Candidatus Jettenia ecosi isolate J2	GCA_005524015.1
Candidatus Brocadia sp. WS118	GCA_007618135.1
Candidatus Kuenenia stuttgartiensis isolate BL10	GCA_007618145.1
Candidatus Brocadia sp. BL1	GCA_007618155.1
Candidatus Brocadia sp. AMX2	GCA_008363395.1
Candidatus Jettenia sp. AMX1	GCA_008363445.1
Candidatus Scalindua sp. isolate amx 105	GCA_008501815.1
Candidatus Scalindua sediminis isolate MohnsGC08_192	GCA_008636105.1
Candidatus Brocadia sp. isolate SB37	GCA_008933285.1
Candidatus Scalindua sp. isolate HyVt-343	GCA_011050115.1
Candidatus Kuenenia stuttgartiensis strain CSTR1	GCA_011066545.1
Candidatus Kuenenia sp. isolate AMX2	GCA_011525635.1
Candidatus Brocadia sp. isolate AMX1	GCA_011525665.1
Candidatus Brocadia sp. isolate AMX3	GCA_011526495.1
Candidatus Scalindua sp. isolate GLR123	GCA_013139855.1
Candidatus Brocadia sp. isolate MAG_8	GCA_013360945.1
Candidatus Brocadia sinica isolate MAG_7	GCA_013360995.1
Candidatus Brocadia sp. isolate AMX2	GCA_014338225.1
Candidatus Jettenia sp. isolate AMX1 27	GCA_014338295.1

Candidatus Brocadia sp. isolate Ega_18-Q3-R5-49	GCA_016699285.1
Candidatus Brocadia sinica BroJett002	GCA_016860665.1
Candidatus Jettenia caeni BroJett041	GCA_016861385.1
Candidatus Scalindua sediminis isolate Bin_192	GCA_017368835.1
Candidatus Scalindua arabica isolate SuakinDeep_MAG55_1	GCA_018263435.1
Candidatus Scalindua sp. isolate SI054_bin45	GCA_018655105.1
Candidatus Scalindua sp. isolate SI074_bin13	GCA_018662645.1
Candidatus Scalindua sp. isolate SI075_bin57	GCA_018669395.1
Candidatus Scalindua sp. isolate SI034_bin70	GCA_018675875.1
Candidatus Scalindua sp. isolate SI060_bin37	GCA_018697755.1
Candidatus Scalindua sp. isolate SI072_bin54	GCA_018700855.1
Candidatus Kuenenia stuttgartiensis isolate BROCD040	GCA_019454545.1
Candidatus Brocadia sapporoensis isolate BROCD001	GCA_019455295.1
Candidatus Scalindua rubra isolate MAG_55	GCA_019911885.1
Candidatus Kuenenia stuttgartiensis isolate MAG_32	GCA_019912215.1
Candidatus Kuenenia stuttgartiensis isolate FI88_62_9	GCA_020723505.1
Candidatus Anammoxoglobus sp. isolate DR5_60_7	GCA_020724075.1
Candidatus Brocadia sp. isolate SJ251	GCA_020846915.1
Candidatus Brocadia sp. isolate SJ698	GCA_020851875.1
Candidatus Brocadia sp. isolate SJ692	GCA_020851925.1
Candidatus Brocadia sp. AMX2	GCA_021405165.1
Candidatus Jettenia sp. AMX1	GCA_021405185.1
Candidatus Brocadia sp. isolate DTUAMX1	GCA_021463075.1
Candidatus Brocadia sp. isolate DTUAMX3	GCA_021463785.1
Candidatus Brocadia sp. isolate DTUAMX2	GCA_021463795.1
Candidatus Loosdrechtia aerotolerans isolate YC1	GCA_021646405.1
Candidatus Brocadia sp. isolate YC2	GCA_021646415.1
Candidatus Kuenenia stuttgartiensis isolate YC7	GCA_021646445.1
Candidatus Kuenenia sp. isolate YC6	GCA_021646465.1
Candidatus Brocadia sp. isolate AM9	GCA_021650915.1
Candidatus Scalindua sp. isolate OFTM351	GCA_021776875.1
Candidatus Scalindua sp. isolate OFTM1	GCA_021776915.1
Candidatus Scalindua sp. isolate OFTM256	GCA_021776955.1
Candidatus Scalindua sp. isolate OFTM180	GCA_021777035.1
Candidatus Brocadia sinica HBSIN01	GCA_022180465.1

Candidatus Brocadia sapporoensis HBSAPP01	GCA_022180585.1
Candidatus Jettenia caeni JETCAE04	GCA_022180965.1
Candidatus Kuenenia stuttgartiensis HKUEN01	GCA_022180985.1
Candidatus Scalindua sp. SCALA70	GCA_022181045.1
Candidatus Brocadia sinica isolate MAG.222	GCA_023150675.1
Candidatus Kuenenia stuttgartiensis isolate AMX1	GCA_023384235.1
Candidatus Scalindua sp. isolate Bsp_10	GCA_024223905.1
Candidatus Brocadia sp. isolate AMX2	GCA_024434125.1
Candidatus Jettenia sp. isolate AMX1	GCA_024434255.1
Candidatus Kuenenia sp. isolate S40-7	GCA_024654045.1
Candidatus Scalindua sp. isolate S40-8	GCA_024654085.1
Candidatus Scalindua sp. isolate P6-7	GCA_024654465.1
Candidatus Kuenenia stuttgartiensis isolate kuenenia	GCA_900232105.1
Candidatus Kuenenia stuttgartiensis isolate kuenenia	GCA_900232175.1
uncultured Candidatus Kuenenia sp. isolate AMX1	GCA_900696675.1
Candidatus Loosdrechtia	GCA_21646405.1
Candidatus Brocadia pituitae	GCA_17347445.1

Table SI-5: Percent of genomes covered by reads (breadth) in six metagenomes. Genomes average coverage than less than 10x are reported as having zero breadth.

	**Genome coverage**					
**Genome**	**Sample 1**	**Sample 2**	**Sample 3**	**Sample 4**	**Sample 5**	**Sample 6**
Anammox_bin.281*	0.9998	0.9998	0.9998	0.9998	0.9998	0.9998
Anammox_bin.357*	0.9978	0.9978	0.9978	0.9979	0.9978	0.9978
JAMMSM_AOB_1	0.7252	0.7298	0.7123	0.7354	0.7264	0.7098
JAMMSM_AOB_2	0.6593	0.4998	0.3596	0.4678	0.3377	0.4389
JAMMSM_AOB_3	0.4306	0.3308	0.3521	0.6524	0.4410	0.5426
JAMMSM_AOB_4	0.0000	0.0000	0.0000	0.0000	0.0000	0.0000
JAMMSM_AOB_5	0.0000	0.0000	0.0000	0.0000	0.0000	0.0000
JAMMSM_AOB_6	0.0000	0.0000	0.0000	0.0000	0.0000	0.0000
JAMMSM_AOB_7	0.1161	0.0000	0.0000	0.1327	0.0000	0.1286
JAMMSM_AOB_8	0.0000	0.0000	0.0000	0.0000	0.0000	0.0000
JAMMSM_AOB_9	0.1230	0.0000	0.0000	0.0000	0.0000	0.0000
JAMMSM_CMX_1	0.6658	0.7256	0.8520	0.9112	0.7852	0.8979
JAMMSM_CMX_2	0.3669	0.3130	0.3586	0.4463	0.3149	0.4111
JAMMSM_NOB_2	0.3582	0.3523	0.3523	0.4252	0.3325	0.3888
JAMMSM_NOB_4	0.9994	0.9993	0.9998	0.9999	0.9993	0.9996
JAMMSM_NOB_5	0.3877	0.4411	0.4367	0.5717	0.3877	0.4747
JAMMSM_NOB_6	0.8739	0.9049	0.9317	0.9734	0.8899	0.9476
JAMMSM_NOB_7	0.9271	0.9285	0.9345	0.9664	0.9025	0.9618
Nitrospira_bin.184*	0.9993	0.9995	0.9995	0.9996	0.9995	0.9994
Nitrospira_bin.465*	0.9976	0.9977	0.9977	0.9978	0.9976	0.9977

* Genomes assembled in this study.

Table SI-6: Relative abundances in reads per kilobase million (RPKM) of MAGs with average genome coverage higher than 50%.

	RPKM					
**Genome**	**Sample 1**	**Sample 2**	**Sample 3**	**Sample 4**	**Sample 5**	**Sample 6**
Anammox_bin.281	15.86	14.97	16.17	14.63	13.95	15.25
Anammox_bin.357	8.77	8.29	9.04	8.23	7.83	8.43
JAMMSM_AOB_1	0.48	0.37	0.37	0.37	0.44	0.35
JAMMSM_CMX_1	0.95	0.81	0.83	1.02	0.89	0.92
JAMMSM_NOB_4	3.87	3.97	3.94	6.30	3.66	5.44
JAMMSM_NOB_6	2.69	2.78	2.91	4.38	2.87	3.80
JAMMSM_NOB_7	2.01	1.72	1.73	2.26	1.88	2.01
Nitrospira_bin.184	3.02	3.20	3.39	5.16	3.31	4.47
Nitrospira_bin.465	3.20	3.26	3.40	5.23	3.31	4.43

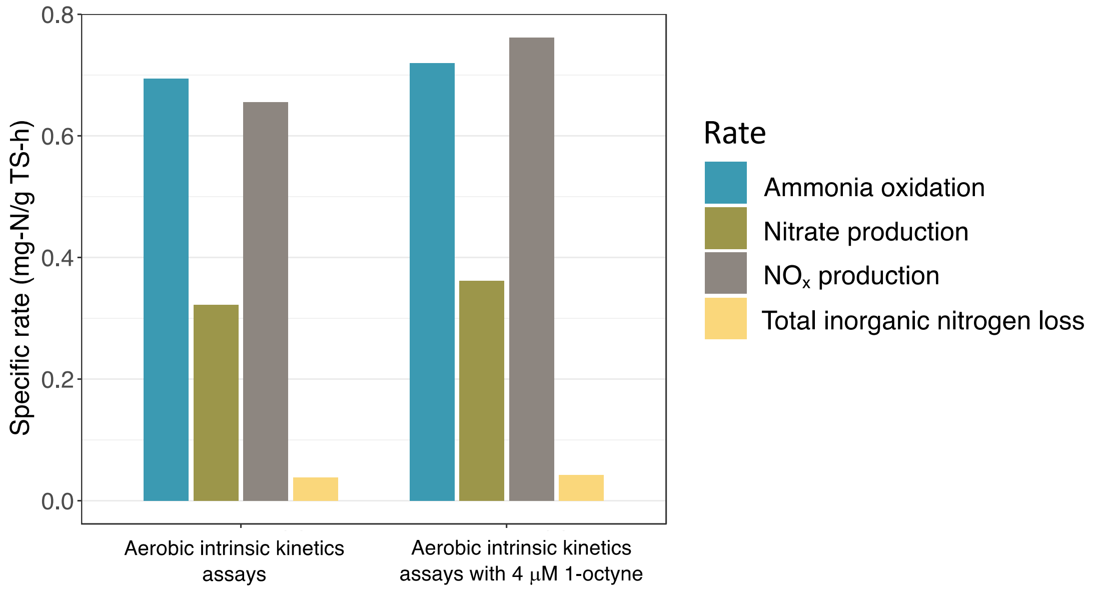

Figure SI-1: Specific rates for ammonia oxidation (blue), NOx production (green), and total inorganic nitrogen loss (yellow) obtained from aerobic ammonia oxidation batch assays for the suspended solids. Bars for each condition are from a single batch assay.

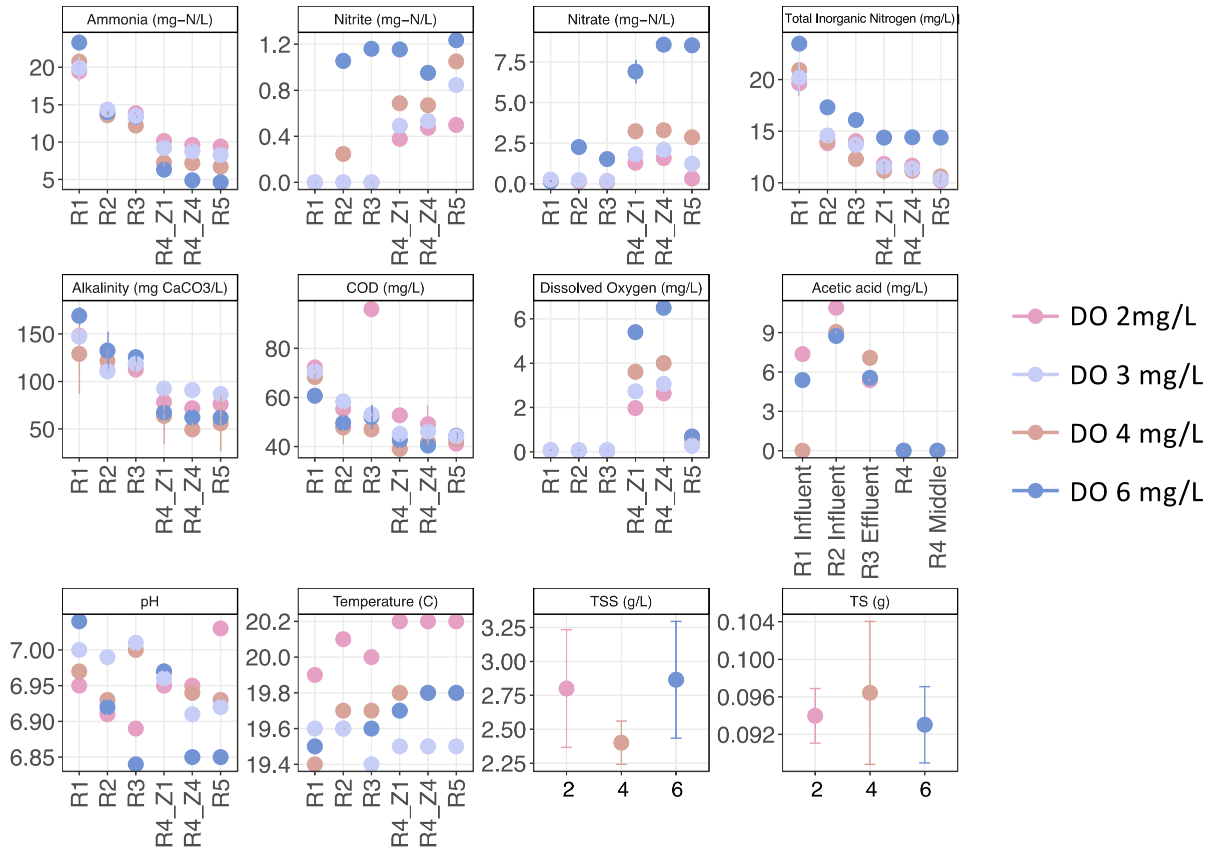

Figure SI-2: Measurements for alkalinity, COD, DO, acetic acid, pH, and temperature sampled along the transect of the IFAS system defined in Section S4 and TSS and TS from the aerobic zone. All samples were taken during full-scale DO set point experiments (2-6 mg/L). Average values were reported for ammonia, nitrite, nitrate, alkalinity, and COD and single values for dissolved oxygen, pH, temperature, and acetic acid. Average values of TSS and TS were reported for the suspended and attached phase. Error bars represent the standard deviations. Influent wastewater contained average ammonia, nitrite and nitrate concentrations of 20.85 (± 1.75), 0 and 0.20 (± 0.04) mg-N/L. Alkalinity, COD, pH, total suspended solids, and total solids measurements were similar in each respective zone for all sampling days. The temperature in the IFAS system was consistent throughout full-scale experiments, ranging from 19.4°C-20.2 °C. Dissolved oxygen concentrations measured in the anoxic zones (R1-R3) were consistently less than 0.07 mg/L, while the desired DO set points in the aerobic zone were achieved for full-scale experiments on their designated days. Slightly higher DO concentrations at the end of the aerobic zone (R4_Z4) were observed compared to the beginning (R4_Z1) but remained within an acceptable range of the DO setpoints. Influent acetic acid concentrations measured on days when the DO set points were 2 and 6 mg/L were 7.37 and 5.39 mg/L, respectively, but were below the LOQ on the day the DO was set to 4 mg/L. Given the detected influent concentrations are close to the LOQ, its possible acetic acid was also present in the influent when the DO was set to 4 mg/L. Across the anoxic zone (R1-R3), acetic acid was produced and then consumed but was not further detected in the aerobic zone.

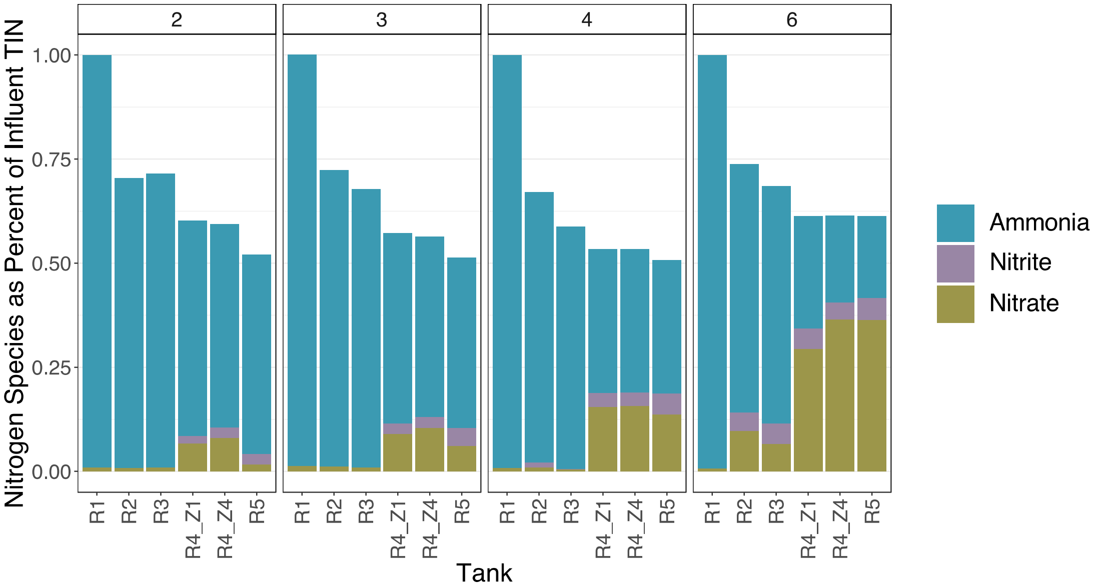

Figure SI-3: Nitrogen species as a percent of influent total inorganic nitrogen (TIN) along the transect of the IFAS system. The facets are labelled based on the DO setpoints. Bars were generated by taking the average concentrations of ammonia (navy), nitrite (orange), and nitrate (green) between sample duplicates and dividing them by the average concentration of influent total inorganic nitrogen. This was done for each sampling location at all DO settings (2-6 mg/L). The average concentration of influent TIN was the summation of ammonia, nitrite, and nitrate concentrations acquired from sample duplicates at R1. Profiles of inorganic nitrogen indicated similar compositions of nitrogen species (mostly as ammonia) entering the aerobic zone when the DO was set to 2, 3, and 4 mg/L, but a higher proportion of nitrite and nitrate entered the zone when the DO was set in 6 mg/L. In this case, nitrate production in the aerobic zone was the highest out of all DO set points, and as a result, more nitrate was returned to the anoxic zone (R2/R3) through the internal recycle line. A dilution effect was observed between R1 and R2 on each sampling day due to the internal recycle coming into the anoxic zone at R2. For example, the average influent ammonia concentration (R1) on one sampling day was 19.45 mg NH_3-N/L, and it was diluted to 13.7 mg NH_3-N /L in R2.

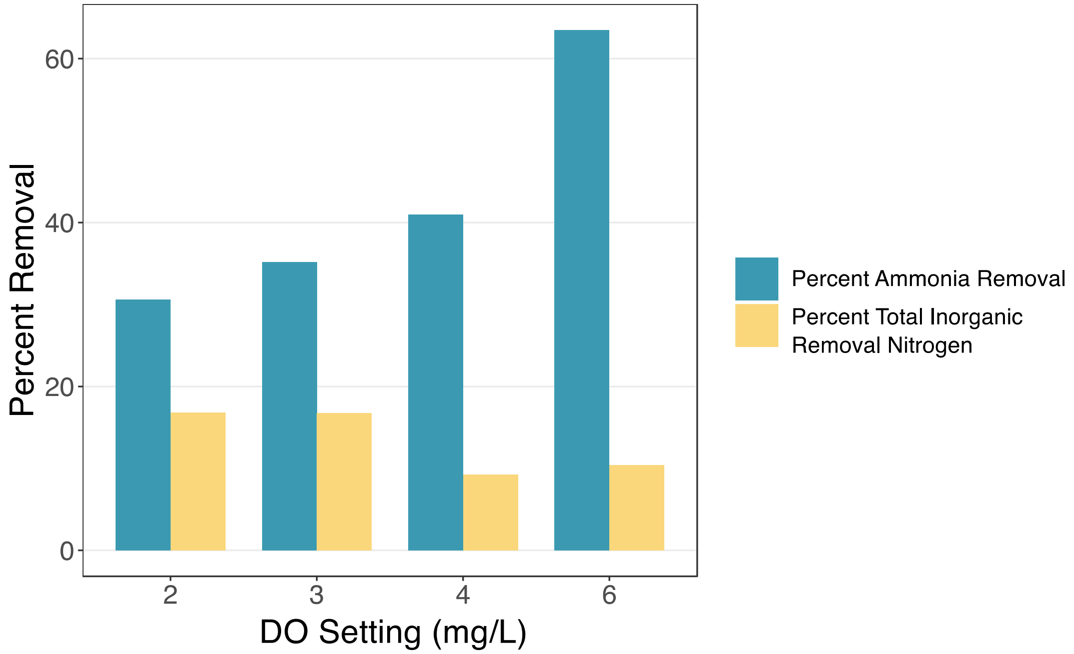

Figure SI-4: Percent of ammonia (navy) and total inorganic nitrogen (yellow) removal in the aerobic zone at each DO set point. Percent removal is calculated from average ammonia or total inorganic nitrogen aerobic zone influent concentration (Figure 1, R3) minus aerobic zone effluent concentration (R4_Z4) divided by the aerobic zone influent concentration.

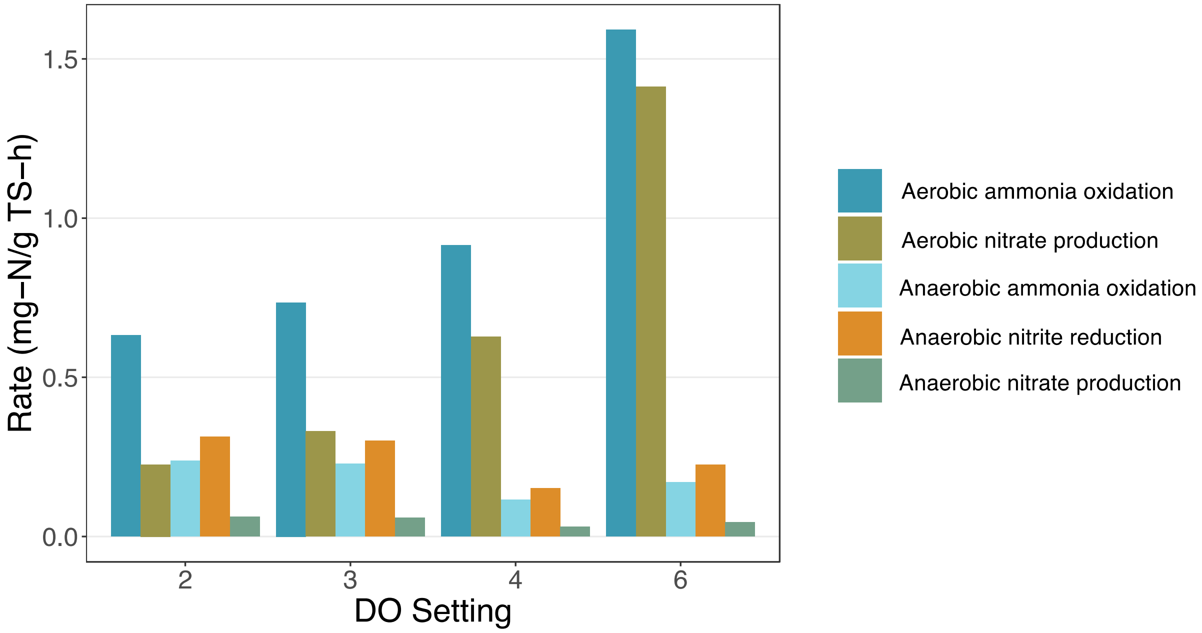

Figure SI-5: Specific rates for aerobic and anaerobic nitrogen biotransformations in the aerobic zone of the full-scale IFAS system during DO set point experiments. Rates were calculated using equations defined in Section S4.
